# Inhibition of Proliferation and Induction of Apoptosis in Prostatic Carcinoma DU145 Cells by Polysaccharides from Yunnan *Rosa roxburghii* Tratt

**DOI:** 10.3390/molecules29071575

**Published:** 2024-04-01

**Authors:** Ziyan Yang, Guiyuan Chen

**Affiliations:** School of Basic Medicine, Dali University, Dali 671003, China; zyyang9991@163.com

**Keywords:** prostate cancer, extraction and decolorization of *Rosa roxburghii* Tratt polysaccharide, cell cycle, cell apoptosis

## Abstract

Objective: This study aimed to investigate methodologies for the extraction and purification of polysaccharides from *Rosa roxburghii* Tratt fruits and their impact on various cellular processes in prostate cancer DU145 cells, including survival rate, migration, invasion, cell cycle, and apoptosis. Results: Compared to the control group, the polysaccharide exhibited a significant reduction in the viability, migration, and invasion rates of DU145 cells in a time- and dose-dependent manner within the polysaccharide-treated groups. Additionally, it effectively arrested the cell cycle of DU145 cells at the G0/G1 phase by downregulating the expressions of CDK-4, CDK-6, and Cyclin D1. Furthermore, it induced apoptosis by upregulating the expressions of Caspase 3, Caspase 8, Caspase 9, and BAX. Methods: Polysaccharides were extracted from *Rosa roxburghii* Tratt sourced from Yunnan, China. Extraction and decolorization methods were optimized using response surface methodology, based on a single-factor experiment. Polysaccharide purification was carried out using DEAE-52 cellulose and Sephadex G-100 column chromatography. The optimal dosage of *R. roxburghii* Tratt polysaccharide affecting DU145 cells was determined using the CCK-8 assay. Cell migration and invasion were assessed using transwell and scratch assays. Flow cytometry was employed to analyze the effects on the cell cycle and apoptosis. Western blotting and Quantitative real-time PCR were utilized to examine protein and mRNA expressions in DU145 cells, respectively. Conclusions: *Rosa roxburghii* Tratt polysaccharides, consisting of D-mannose, L-rhamnose, N-acetyl-D-glucosamine, D-galacturonic acid, D-glucose, D-galactcose, D-xylose, L-arabinose, and L-fucose, possess the ability to hinder DU145 cell proliferation, migration, and invasion while inducing apoptosis through the modulation of relevant protein and gene expressions.

## 1. Introduction

Cancer is the leading cause of death and a significant barrier to increasing life expectancy globally [1]. Among urological malignancies, prostate, bladder, and kidney cancers stand out as primary contributors to morbidity and mortality in males aged over 40 years [2]. Prostate cancer ranks as the second most prevalent cancer among men and represents the fifth leading cause of cancer-related deaths worldwide [3]. Prostate cancer represents a prevalent global health issue, with an anticipated rise in its incidence [4]. Current approaches to managing prostate cancer predominantly encompass radiotherapy and chemotherapy, which not only incur substantial costs but also carry a burden of adverse effects. DU145 cells, derived from human prostate cancer epithelium, exhibit robust proliferative capabilities, capable of undergoing division within 48 h under optimal growth conditions. Additionally, prostate cancer is distinguished by its prolonged course and propensity for metastasis.

Cells serve as the fundamental units of life, playing indispensable roles in maintaining life, facilitating reproduction, and adapting to environmental changes. During organismal growth and development, the processes of cell proliferation and death persist, with the cell cycle and apoptosis serving as critical mechanisms regulating these phenomena, respectively. Even cancer cells adhere to cell cycles, and numerous cytotoxic agents aim to disrupt different stages of these cycles in cancer cells to impede their division, consequently exerting anti-tumor effects.

Progression through the cell division cycle is driven by cyclins, which bind to and activate their catalytic partners, the cyclin-dependent kinases (CDKs) [5]. CDKs, along with their binding partner cyclins, regulate cellular growth across the cell cycle phases [6]. Among these regulators, CDK-4 and CDK-6 play pivotal roles and are frequently dysregulated in various human cancers [7]. CDK-4 plays a crucial role in orchestrating the orderly progression of the cell cycle by binding to cyclin D to facilitate the G1/S transition [8]. The cell cycle, a highly conserved and sequential process, includes four phases: G1 (pre-DNA synthesis), S (DNA synthesis), G2 (pre-division), and M (cell division). Transitions between these phases are regulated by diverse CDKs in collaboration with their partner cyclins, ensuring smooth progression through the cell cycle [9]. The cyclin D–CDK-4/-6 complex, pivotal for the G1–S transition, serves as a target for inhibitors that impede the transition from G1 to S phase, thereby halting cell cycle progression [10]. CycD–CDK-4/-6 activation stimulates mTORC1, promoting cell proliferation through Retinoblastoma (RB) phosphorylation and stimulation of cell growth [11]. Expression levels of CDK-4/-6 are notably elevated in various human cancers, including ovarian cancer, where its expression is inversely related to patient prognosis [12]. The malignant proliferation of tumor cells is characterized by dysregulated cell cycle and apoptosis regulation [13]. Studies showed that *gypenoside LI* exhibits the ability to inhibit the proliferation and migration of human breast cancer cells, induce apoptosis, and arrest the cell cycle at the G0/G1 phase by modulating E2F1 [14]. Fann and colleagues found that treatment with *anthraquinone* (*CC12*) resulted in reduced Bcl2 protein expression in two glioblastoma cell lines, 87MG and U118MG, suggesting that CC12 induces apoptosis in tumor cells and arrests cells in the G1 phase [15]. 

Apoptosis, or programmed cell death, is pivotal in various biological processes, including embryonic development, tissue remodeling, and cell homeostasis [16]. Apoptosis is the cell’s natural intrinsic regulatory mechanism of normal cells for programmed cell death, which plays an important role in cancer as a classical mechanism of tumor cell death [17]. Organismal development and function requires multiple and accurate signal transduction pathways to ensure that proper balance between cell proliferation, differentiation, inactivation, and death is achieved [18]. Caspases are proteases conserved for initiating and executing the apoptotic program [19]. The cell death pathway, primarily mediated by the caspase family’s proteolytic enzymes, is activated by internal or external stimuli. Caspases are crucial in apoptosis, inflammation, and cell proliferation [20], constituting a family of cysteine proteases responsible for initiating and executing the apoptotic process [21]. *Ivermectin* (*IVM*) down-regulated the expression level of the anti-apoptotic protein Bcl-2 in the cytoplasm of HeLa cells, while the expression of the pro-apoptotic protein Bax exhibited dose-dependent effects [22]. In comparison to the control group, *pterostilbene* (*PTE*) increased the expression levels of pro-apoptotic proteins Bax, Cleaved-Caspase 3, and Cleaved-Caspase 9 in human renal cell carcinoma (RCC) cells and reduced the expression of the anti-apoptotic protein Bcl-2, indicating that PTE’s inhibitory effect on RCC cell growth may be attributed to the induction of apoptosis [23]. Furthermore, CDK-4/-6 inhibitors arrest the cell cycle at the G1 phase and hinder the proliferation of aggressive cells, showing promise in attenuating the aggressiveness of breast cancers [24]. Apoptosis can be triggered via two primary pathways: the extrinsic death receptor pathway and the intrinsic mitochondrial pathway [25].

Oxidative stress due to abnormal accumulation of reactive oxygen species (ROS) is an initiator of a large number of human diseases [26]. A physiological level of oxygen/nitrogen free radicals and non-radical reactive species is termed oxidative stress [27]. OS is a chemical imbalance between an oxidant and an antioxidant, causing damage to redox signaling and control or causing molecular damage. Unbalanced oxidative metabolism can produce excessive reactive ROS. The formation of cancer and its progression is strongly associated with oxidative stress and the resulting oxidative injury [28]. ROS in cancer cells play a central role in regulating and inducing apoptosis, thereby modulating cancer cell proliferation, survival, and drug resistance [29].

In recent decades, polysaccharides derived from various Chinese herbs have garnered significant attention owing to their critical anti-tumor biological activity [30]. At present, cancer treatment predominantly relies on radiotherapy and chemotherapy, methods known for their considerable cost and the myriad adverse effects they inflict on patients’ physical and psychological well-being. Consequently, there has been a notable surge in research focusing on the exploration of anticancer constituents present in natural products. Plants harbor a wealth of active compounds, with plant polysaccharides standing out prominently. Numerous studies have underscored their diverse array of activities, encompassing antiviral, anti-tumor, anti-aging, radioprotective, anti-stress, and antioxidant properties [31].

Consequently, this study aimed to explore the potential effects of a polysaccharide extracted and decolorized from *Rosa roxburghii* Tratt on the proliferation and apoptosis of DU145 prostate cancer cells.

## 2. Results

### 2.1. Impact of Single Factors on Polysaccharide Extraction Rate in Rosa roxburghii Tratt

#### 2.1.1. Material–Liquid Ratio

**Figure 1:**
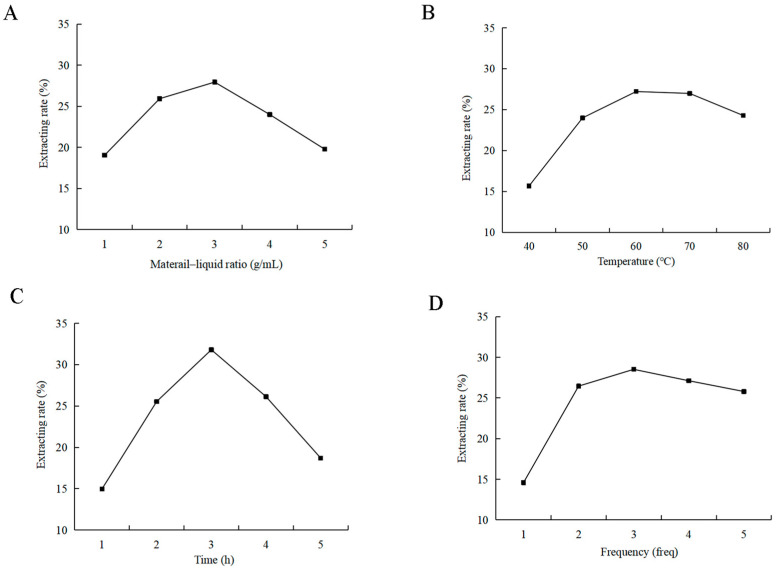
Effects of single factors on the extraction yield of *Rosa roxburghii* Tratt polysaccharide. (**A**): Effect of material–liquid ratio on polysaccharide extraction yield; (**B**) effect of extraction temperature on polysaccharide extraction yield; (**C**) effect of extraction time on polysaccharide extraction yield; (**D**) effect of extraction frequency on polysaccharide extraction yield.

Under the extraction conditions of 60 °C, time 1 h, and a single extraction cycle, the polysaccharide extraction yield was evaluated across material-to-liquid ratios of 1:10, 1:20, 1:30, 1:40, and 1:50 g/mL. As illustrated in Figure 1A, the highest polysaccharide extraction yield, reaching 27.94%, was attained at an optimal material–liquid ratio of 1:30 g/mL. Above this ratio, the extraction efficiency decreased.

#### 2.1.2. Extraction Temperature

Under the extraction conditions of material-to-liquid ratio of 1:30 g/mL, time 1 h, and single extraction cycle, the polysaccharide extraction yield was assessed at temperatures of 40, 50, 60, 70, and 80 °C. As depicted in Figure 1B, the highest extraction yield, reaching 27.21%, was attained at an optimal temperature of 60 °C. Further increases in temperature led to a reduction in yield.

#### 2.1.3. Extraction Time

Maintaining a constant material-to-liquid ratio of 1:30 g/mL, temperature of 60 °C, and single extraction cycle, the polysaccharide extraction yield was examined across extraction times of 1, 2, 3, 4, and 5 h. As depicted in Figure 1C, the peak extraction yield of 28.01% was achieved at 3 h. Beyond this duration, a decline in extraction yield was observed.

#### 2.1.4. Extracting Frequency

Maintaining the conditions of material-to-liquid ratio of 1:30 g/mL, temperature of 60 °C, and time of 3 h, the polysaccharide extraction yield was evaluated for extraction frequencies of 1, 2, 3, 4, and 5 cycles. As illustrated in Figure 1D, the peak extraction yield of 28.52% was attained with an extraction frequency of 3 times.

#### 2.1.5. Response-Surface Test

Based on the factors and their corresponding levels outlined in Table 1, a response-surface test was conducted using the Box–Behnken design, following the single-factor test. ANOVA results are shown in Table 2, and the results of the error of the regression model are shown in Table 3.

Multivariate binomial regression and multivariate linear analysis were utilized to evaluate the impact of variables *A*, *B*, *C*, and *D* on the response variable of comprehensive score *S*. The regression equation is presented as follows:

*Y* = +29.46 + 0.75*A* + 0.77*B* − 1.09*C* − 0.97*D* − 0.040*AB* + 0.26*AC* − 0.080*AD* − 0.61*BC* − 0.22*BD* − 0.52*CD* − 2.23*A*^2^ − 0.71*B*^2^ − 1.42*C*^2^ − 1.77*D*^2^

Table 2 and Table 3 illustrate that the F-test of the model yields *p* < 0.0001, indicating high significance of the regression equation and a well-fitted regression region for the model. The lack-of-fit item exhibits a *p*-value of 0.0915 (*p* > 0.05), suggesting that the difference in the regression equation is not significant, thus affirming the good fit of the binomial multinomial regression equation. With a coefficient of variation (C.V.%) of 2.79%, there is minimal dispersion among the variables, implying higher reliability of the regression model when the C.V.% is lower. The R-squared (*R*^2^) value of 0.9106 and the adjusted R-squared (*R*^2^_Adj_) value of 0.8212 indicate a robust linear correlation between variables and minimal experimental error. The signal-to-noise ratio, or adequate precision, stands at 11.3, implying a robust model fit, as values above 4 are generally considered satisfactory. Furthermore, Table 1 reveals that factors *A*, *B*, *C*, *D*, *A*^2^, *C*^2^, and *D*^2^ significantly or highly significantly impact the polysaccharide extraction yield. These analytical outcomes validate the applicability of the model for analyzing and predicting the extraction parameters of total polysaccharides from *Rosa roxburghii* Tratt using water extraction and alcohol precipitation.

#### 2.1.6. Verification Test

Design-Expert 13 predicted the optimal extraction conditions as follows: a material–liquid ratio of 1:25 g/mL, an extraction temperature of 68.01 °C, an extraction time of 3.14 h, and an extraction frequency of 2.74. For practical application, adjustments were made to a material–liquid ratio of 1:30 g/mL, an extraction time of 3 h, an extraction temperature of 70 °C, and an extraction frequency of three times. Three parallel experiments were conducted. The polysaccharide extraction rate was 30.56 ± 0.89%, which is close to the predicted value of 30.22%, with an error of 1.12%. These results confirm that the optimized extraction technique for *Rosa roxburghii* Tratt polysaccharide using the response surface methodology is stable and viable.

### 2.2. Structural Characterization of RTDP

#### 2.2.1. Isolation and Purification of RTDP

**Figure 2:**
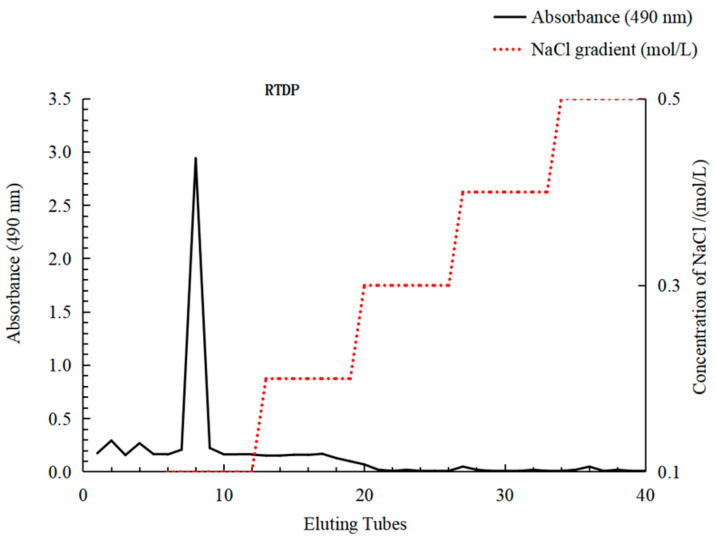
The eluting curve of the *Rosa roxburghii* Tratt decolorized polysaccharide (*RTDP*).

As depicted in Figure 2, the elution profile exhibited a single distinct peak, representing the water-eluted polysaccharide *RTDP*. To gather enough polysaccharide for activity analysis and structural determination, the water-eluted polysaccharide underwent further purification.

#### 2.2.2. Analysis of Molecular Weight and Monosaccharide Composition

**Figure 3:**
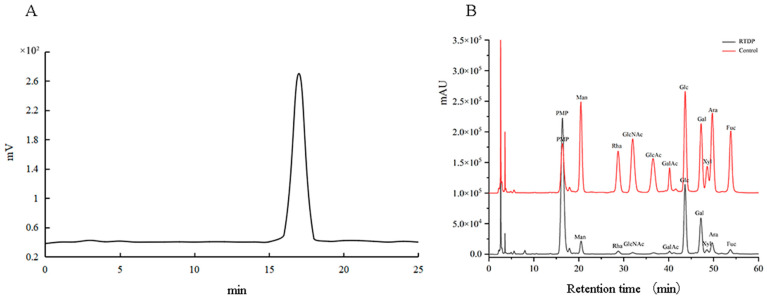
Molecular weight and monosaccharide composition of *RTDP.* (**A**) The HPGPC chromatograms of RTDP; (**B**) The HPLC chromatograms of RTDP.

The molecular weight of *RTDP* was determined using the High performance gel permeation chromatography (HPGPC) method. Figure 3A illustrates a unimodal distribution of *RTDP*, indicating its polysaccharide homogeneity. The principal molecular weight peak, observed at 2.7 × 10^2^ kDa, was identified based on retention time.

As depicted in Figure 3B, the hydrolyzed *RTDP* exhibited nine significant absorption peaks. The molar ratio of monosaccharides was determined by High Performance Liquid Chromatography (HPLC) method as follows: Man:Rha:N-Acetyl-Glc:D-Gal-UA:D-Glc:D-Gal:D-Xyl:L-Ara:L-Fuc = 3.95:2.51:1.17:1.00:26.59:13.65:1.29:8.62:7.89. This analysis indicates that *RTDP* consists of these nine monosaccharides. Notably, D-Glc was found to be the main component, suggesting that *RTDP* is an acidic polysaccharide.

#### 2.2.3. FT-IR and Ultraviolet–Visible Spectroscopy

**Figure 4:**
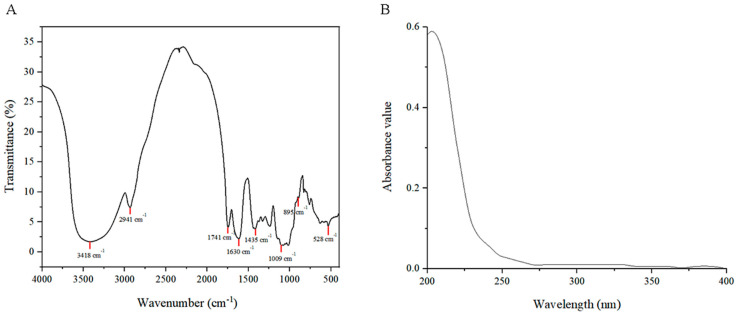
FT−IR and UV–vis spectrum of *RTDP*. (**A**) The FT–IR spectra of *RTDP*; (**B**) The UV spectra of *RTDP*.

As shown in Figure 4A, Fourier Transform Infrare (FT-IR) spectroscopy revealed a prominent absorption peak at 3418 cm^−1^ in *RTDP*, attributed to the O–H stretching vibration. Additionally, a methylene (–CH_2_) stretching vibration absorption peak appeared at 2941 cm^−1^. The peak at 1741 cm^−1^ was associated with the stretching vibration of carboxylate groups, while peaks at 1009 cm^−1^ and 1421 cm^−1^ corresponded to the stretching vibrations of C–O–C. These results suggest that *RTDP* is an acidic polysaccharide rich in carboxylic acid groups, consistent with the monosaccharide composition analysis.

Furthermore, Figure 4B indicates that *RTDP* does not contain proteins and nucleic acid because there are no characteristic absorption peaks at 260 and 280 nm in the Ultraviolet–Visible (UV) spectrum.

#### 2.2.4. Congo Red Test

**Figure 5:**
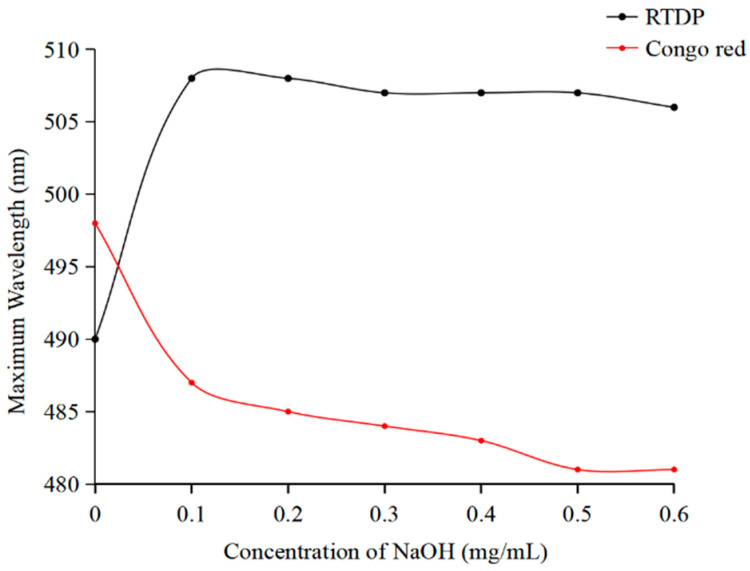
Maximum absorption wavelengths of Congo red mixed with *RTDP* at various concentrations of NaOH.

Congo red interacts with polysaccharides characterized by a triple helix structure, inducing a redshift in the maximum absorption wavelength compared to Congo red solution alone [32]. UV–Vis spectroscopy analysis revealed that the maximum absorption wavelength of *RTDP* is redshifted compared to Congo red, indicating the presence of a triple helix structure in *RTDP* (Figure 5).

#### 2.2.5. Effects of Decolorization Factors on the Decolorization Rate and Polysaccharide Retention Rate of *Rosa roxburghii* Tratt Polysaccharide

**Figure 6:**
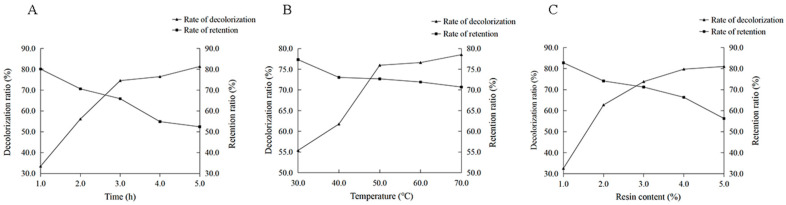
Effect of each factor on the decolorization rate and retention rate of *RTDP*. (**A**) Effect of decolorization time on the decolorization rate and retention rate of *RTDP*; (**B**) effect of macroporous resin content on the decolorization rate and retention rate of *RTDP*; (**C**) effect of decolorization temperature on the decolorization rate and retention rate of *RTDP*.

An escalation in decolorization time resulted in an augmented decolorization rate but a diminished polysaccharide retention rate. A time of 2 h was determined as optimal for subsequent single-factor tests. Notably, the polysaccharide retention rate exhibited a significant decline beyond 3 h (Figure 6A). The decolorization rate increased with temperature, while the polysaccharide retention rate showed a declining trend. We selected 50 °C as the optimal temperature for the single-factor experiment (Figure 6B). To minimize polysaccharide loss while ensuring effective decolorization, 3 g of resin was selected for the subsequent experiments (Figure 6C).

### 2.3. Results of the Response Surface Optimization Methodology

#### 2.3.1. Response Surface Test Design and Results

The response surface test, conducted using the Box–Behnken design, utilized the factors and levels (Table 4) based on a single-factor test. Discrepancies within the regression model are detailed in Table 5, with additional insights into the model’s error provided in Table 6.

#### 2.3.2. Establishment of Fitting Model and Data Analysis

Design Expert 13.0 was employed to analyze the data presented in Table 7, yielding a fitting equation relating the comprehensive score (*S*) with decolorization time (*A*), decolorization temperature (*B*), and macroporous resin content (*C*):

*S* = 72.41 + 0.81*A* + 1.34*B* + 1.3*C* − 1.34*AB* − 1.79*AC* − 2.79*BC* − 2.91*A*^2^ − 8.02*B*^2^ − 7.94*C*^2^

Table 5 indicates that *F* = 70.43 and *p* < 0.0001 for the regression model, signifying its high significance. The R-squared (*R*^2^) value was 0.9891, with an adjusted R-squared (*R*^2^_Adj_) of 0.9750, indicating that the model fits well. In Table 6, the factors *B*, *C*, *A*^2^, *B*^2^, and *C*^2^ significantly influenced the comprehensive score (*p* < 0.05), indicating that these factors significantly impact the decolorization outcome. The interaction terms *AC* and *AB* were significant (*p* < 0.05), while the interaction term *BC* was highly significant (*p* < 0.01), indicating a substantial interaction between decolorization temperature (*B*) and macroporous resin content (*C*). As Table 1, the *F* values for *A*, *B*, and *C* were 4.7, 12.7, and 12.37, respectively, indicating the hierarchy of influence on the comprehensive score as *B* = *C* > *A*.

#### 2.3.3. The Interaction Effect of Each Factor on the Comprehensive Score of Decolorizing Rate and Polysaccharide Retention Rate from RTDP

**Figure 7:**
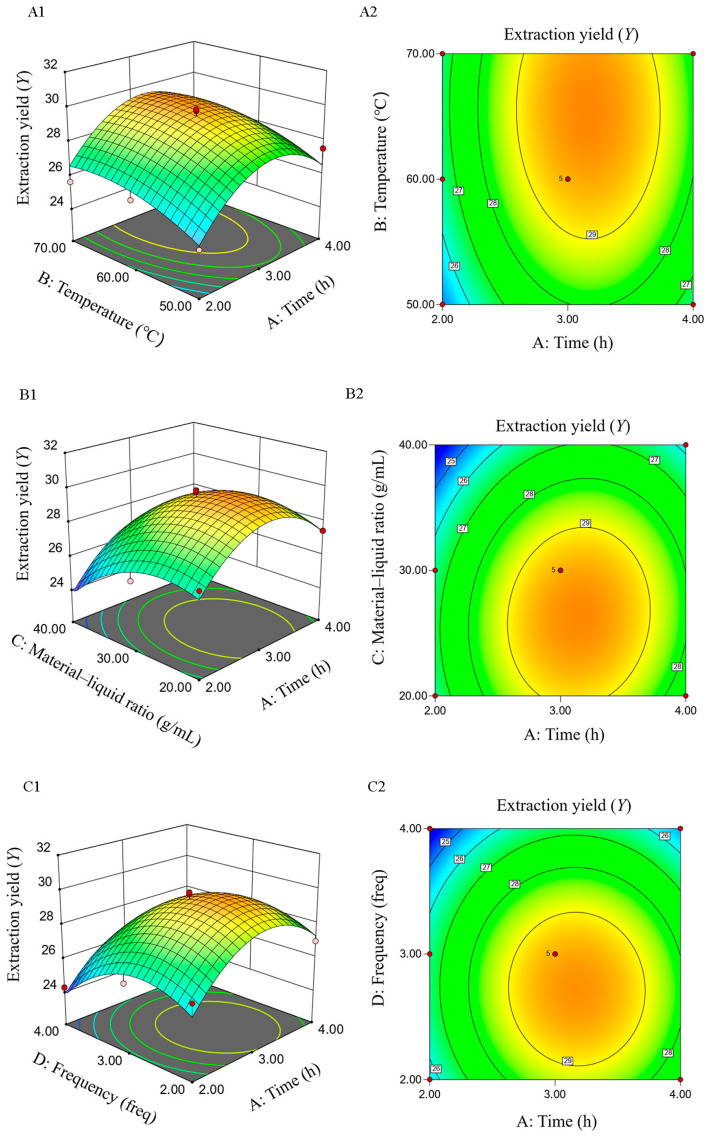

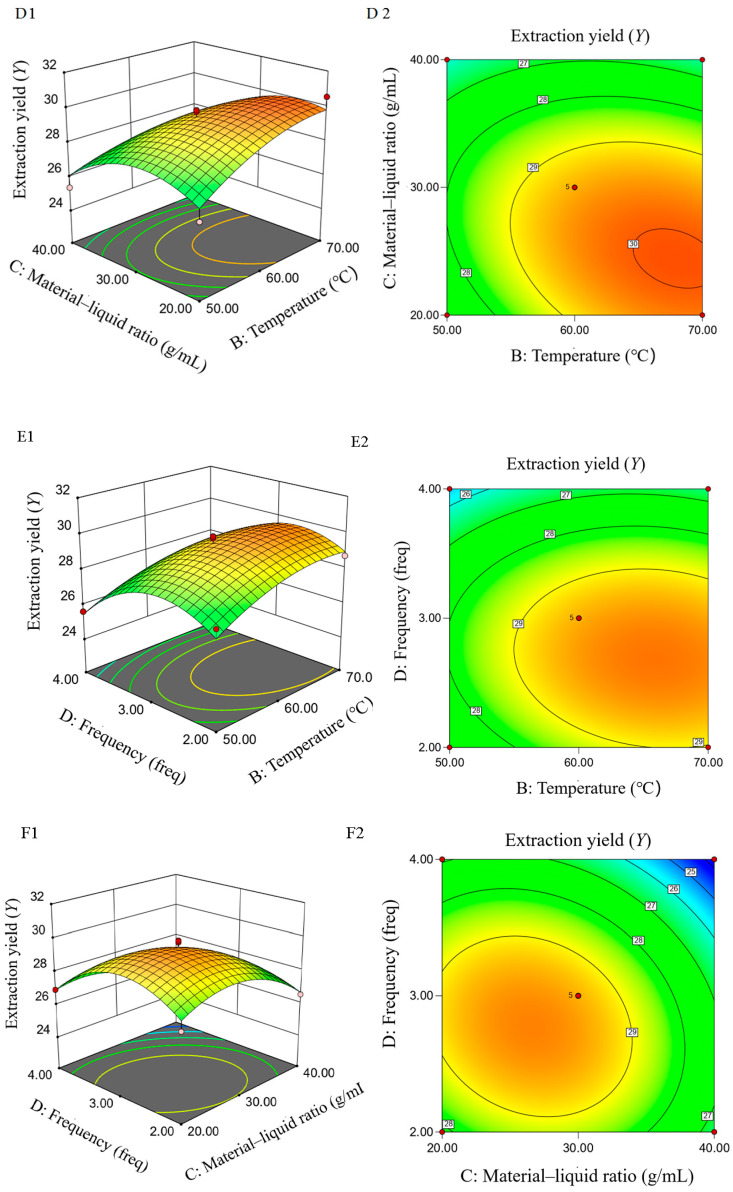
Interaction effect of each factor on the extraction yield *Y* from *RTDP*. (**A**) Effect of time and temperature on the extraction yield of *RTDP* (A1 The 3D surface of the intersection of time and temperature; A2 The contour of the intersection of time and temperature); (**B**) effect of time and material–liquid ratio on the extraction yield of *RTDP* (B1 The 3D surface of the intersection of time and material–liquid ratio; B2 The contour of the intersection of time and material–liquid ratio); (**C**) effect of time and frequency on the extraction yield of *RTDP* (C1 The 3D surface of the intersection of time and frequency; C2 The contour of the intersection of time and frequency); (**D**) effect of temperature and material–liquid ratio on the extraction yield of *RTDP* (D1 The 3D surface of the intersection of temperature and material–liquid ratio; D2 The contour of the intersection of temperature and material–liquid ratio); (**E**) effect of temperature and frequency on the extraction yield of *RTDP* (E1 The 3D surface of the intersection of temperature and frequency; E2 The contour of the intersection of temperature and frequencye); (**F**) effect of material–liquid ratio and frequency on the extraction yield of *RTDP* (F1 The 3D surface of the intersection of material–liquid ratio and frequency; F2 The contour of the intersection of material–liquid ratio and frequency).

**Figure 8:**
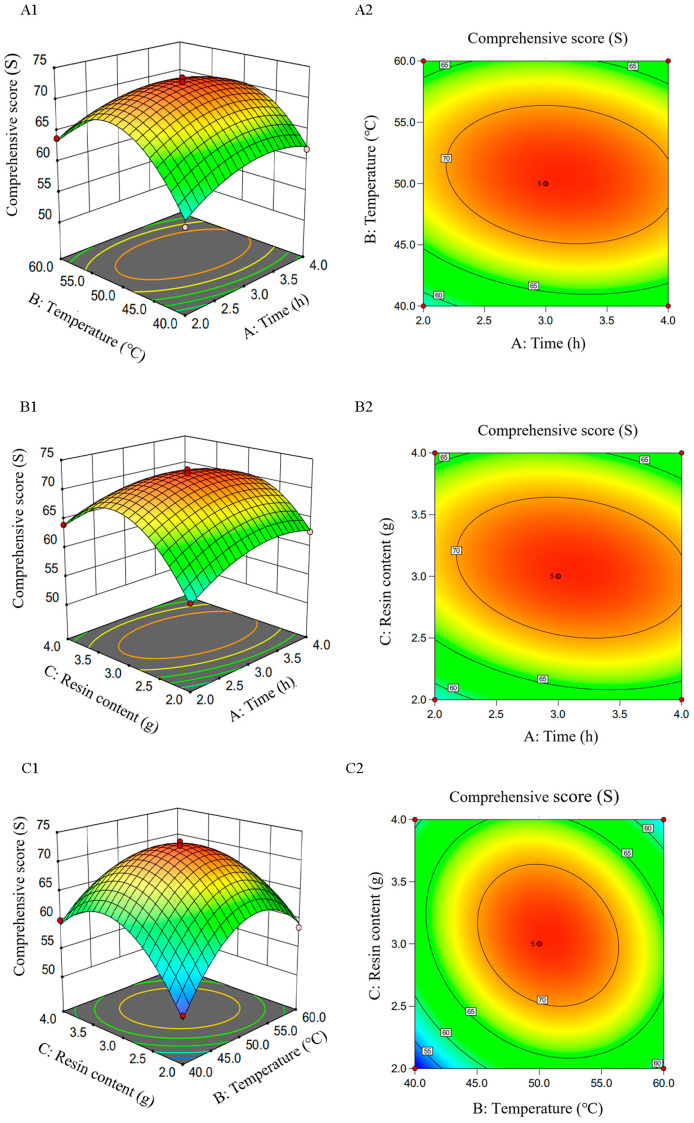
Effect of each factor on the comprehensive score *S* of decolorization rate and retention rate of *RTDP*. (**A**) Effect of decolorization time and temperature on the comprehensive score of *RTDP* (A1 The 3D surface of the intersection of time and temperature; A2 The contour of the intersection of time and temperature); (**B**) effect of decolorization time and resin content on the comprehensive score of *RTDP* (B1 The 3D surface of the intersection of time and resin content; B2 The contour of the intersection of time and resin content); (**C**) effect of decolorization temperature and resin content on the comprehensive score of *RTDP* (C1 The 3D surface of the intersection of temperature and resin content; C2 The contour of the intersection of temperature and resin content).

As shown in Figure 7 and Figure 8, a contour closer to an ellipse indicates a stronger interaction between the two factors, whereas a more circular shape denotes a weaker interaction. In the 3D response surface figure, the gradient of the surface indicates the variation in response value; a steeper surface signifies a more pronounced effect on the response value and vice versa. Figure 8 indicates a significant interaction between *B* and *C*. These findings are in accordance with the regression equation and ANOVA results.

### 2.4. Verification Test

The optimal decolorizing parameters of *RTDP* were predicted by Design-Expert 13 software. Subsequently, a decolorization experiment was conducted using these parameters. The error between the predicted results and the actual values was small (0.59%), demonstrating the stability and reliability of the decolorization technology, indicating it has a good fit.

### 2.5. Detection of the OH^−^ Scavenging Activity of Rosa roxburghii Tratt Polysaccharide

**Figure 9:**
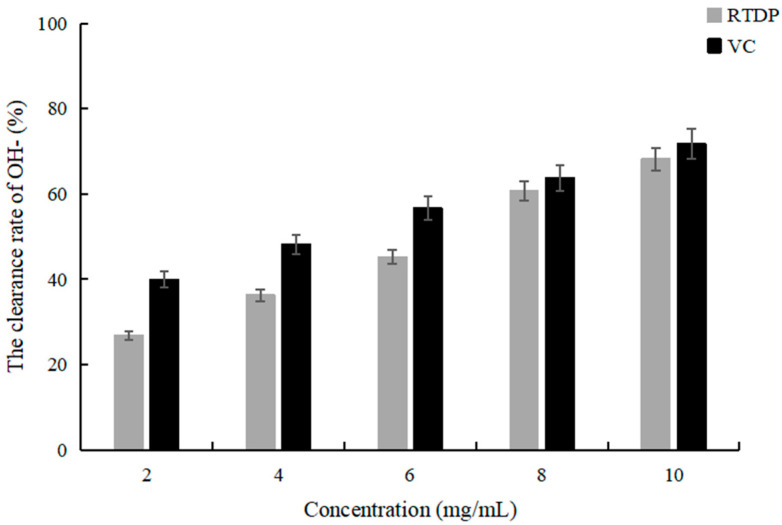
The OH^−^ scavenging activity of *RTDP* and VC.

Reactive oxygen species (ROS) are by-products of normal cellular metabolism [32]. Antioxidants are valuable for their ability to donate electrons, thereby neutralizing radical production and mitigating further damage through free-radical mechanisms [33]. While ascorbic acid is renowned for its antioxidative properties, improper clinical administration may lead to adverse effects such as excessive gastric acid, urinary calculi, and gastrointestinal reactions. Moreover, prolonged excessive consumption can disrupt the body’s regulatory mechanism for vitamin C, potentially resulting in symptoms resembling scurvy [34]. According to Figure 9, the OH^−^ scavenging capacity of *RTDP* at the same concentration was slightly lower than that of VC, but it still showed good oxidation resistance.

### 2.6. DU145 Cells Affected by RTDP

#### 2.6.1. RTDP Decreased the Survival Rate of DU145 Cells

**Figure 10:**
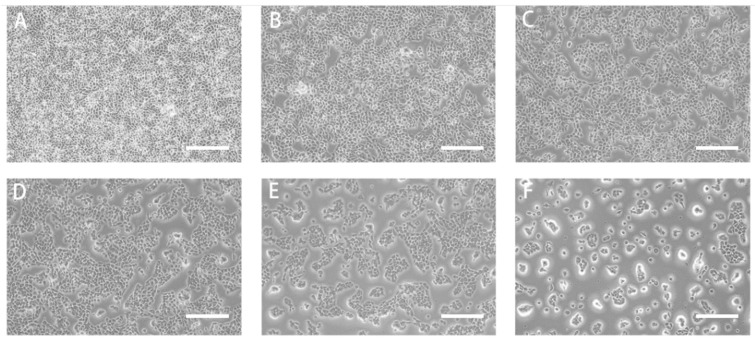
Effect of *Rosa roxburghii* Tratt polysaccharide on the viability of prostate cancer DU145 cells after treatment at 48 h (scale bars = 100 µm): (**A**) 0 mg/mL *RTDP* (control group), (**B**) 2 mg/mL *RTDP*, (**C**) 4 mg/mL *RTDP*, (**D**) 6 mg/mL *RTDP*, (**E**) 8 mg/mL *RTDP*, (**F**) 10 mg/mL *RTDP*.

The absorbance at 450 nm of DU145 cells treated with *RTDP* for durations of 24 h, 48 h, and 72 h was quantified. Figure 10 reveals that, compared to the control group, the survival rate (%) of DU145 cells treated with 2 mg/mL *RTDP* was 99.12 ± 0.67 at 24 h (*p* > 0.05), indicating no significant difference. At 48 h, a decreasing trend in the survival rate of DU145 cells was evident with increasing *RTDP* concentrations. Specifically, the survival rates at different *RTDP* concentrations were as follows: 82.49 ± 0.55 (*p* < 0.01) at 2 mg/mL; 60.92 ± 0.42 (*p* < 0.01) at 4 mg/mL; 51.57 ± 0.68 (*p* < 0.01) at 6 mg/mL; 45.79 ± 1.46 (*p* < 0.01) at 8 mg/mL; and 30.78 ± 0.06 (*p* < 0.01) at 10 mg/mL. These findings indicate a progressive decrease in survival rates with escalating *RTDP* concentrations and duration, indicating *RTDP*’s inhibition of DU145 prostate cancer cell proliferation in a concentration- and time-dependent manner. At 48 h, *RTDP* exhibited a significant inhibitory effect on DU145 cells, with an IC50 of 6.22 mg/mL. Subsequent experiments aimed to determine if *RTDP*’s effect on DU145 cells was dose-dependent, with concentrations of 2 mg/mL (1/3 IC50), 4 mg/mL (2/3 IC50), and 6 mg/mL (IC50), respectively.

#### 2.6.2. RTDP Inhibited the Migration of DU145 Cells

The scratch assay was utilized to assess the migratory capacity of DU145 cells. Compared with the control group, escalating concentrations of *RTDP* led to diminished wound-healing capability within the same treatment duration, suggesting a concentration-dependent effect of *RTDP* on healing ability. Moreover, the duration of *RTDP* treatment showed an inverse correlation with the mobility of the cells.

**Figure 11:**
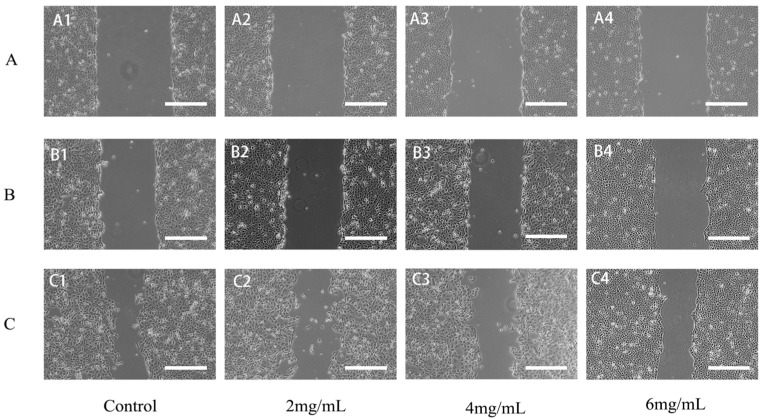
Effect of *Rosa roxburghii* Tratt polysaccharide on the migration of prostate cancer DU145 cells (scale bars = 100 µm). (**A1**–**A4**) DU145 cells were affected by 0, 2, 4, 6 mg/mL *RTDP* for 0 h; (**B1**–**B4**) DU145 cells were affected by 0, 2, 4, 6 mg/mL *RTDP* for 24 h; (**C1**–**C4**) DU145 cells were affected by 0, 2, 4, 6 mg/mL *RTDP* for 48 h.

As shown in Figure 11, the migration rate (%) of DU145 cells treated with *RTDP* for 24 h was 62% ± 0.04 in the control group, 52% ± 0.02 in the 2 mg/mL *RTDP* group (*p* < 0.05), 45% ± 0.02 in the 4 mg/mL *RTDP* group (*p* < 0.01), and 21% ± 0.05 in the 6 mg/mL *RTDP* group (*p* < 0.01). After 48 h, the migration rates were 50% ± 0.07 in the control group, 35% ± 0.05 in the 2 mg/mL group (*p* < 0.05), 26% ± 0.02 in the 4 mg/mL group (*p* < 0.01), and 2% ± 0.01 in the 6 mg/mL group (*p* < 0.01). Comparative analysis revealed that *RTDP* significantly inhibited DU145 cell migration at both 24 and 48 h, with the inhibitory effect intensifying in a time- and dose-dependent manner.

#### 2.6.3. RTDP Inhibited the Invasion of DU145 Cells

**Figure 12:**
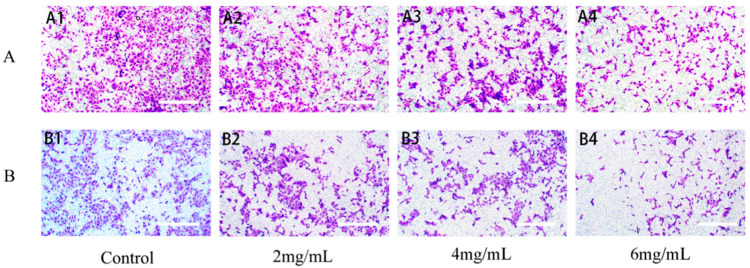
Effect of RTPD on the invasion of DU145 cells (scale bars = 100 µm). (**A1**–**A4**) DU145 cells were treated with 0, 2, 4, and 6 mg/mL *RTDP* for 24 h; (**B1**–**B4**) DU145 cells were treated with 0, 2, 4, and 6 mg/mL *RTDP* for 48 h.

Figure 12 reveals a progressive reduction in the ability of DU145 cells to traverse the polycarbonate membrane following treatment with varying concentrations of *RTDP* compared to the control group. The results indicated an invasion rate at 24 h of 100.51% ± 0.77% in the control group; 78.43% ± 0.47% (*p* < 0.05) in the 2 mg/mL *RTDP* group; 53.51% ± 1.67% (*p* < 0.01) in the 4 mg/mL group; and 31.61% ± 1.59% (*p* < 0.01) in the 6 mg/mL group. The rate of invasion at 48 h was 100.67% ± 0.95% for the control group; 69.39% ± 0.15% (*p* < 0.01) for the 2 mg/mL group; 46.50% ± 0.15% (*p* < 0.01) for the 4 mg/mL group; and 21.57% ± 0.47% (*p* < 0.01) for the 6 mg/mL group. The findings demonstrate that different concentrations of *RTDP* can inhibit DU145 cell invasion, with the inhibitory effect becoming more pronounced as the *RTDP* concentration increases, consequently reducing the number of invasive cells.

#### 2.6.4. RTDP Inhibited the Cell Cycle of DU145 Cells

**Figure 13:**
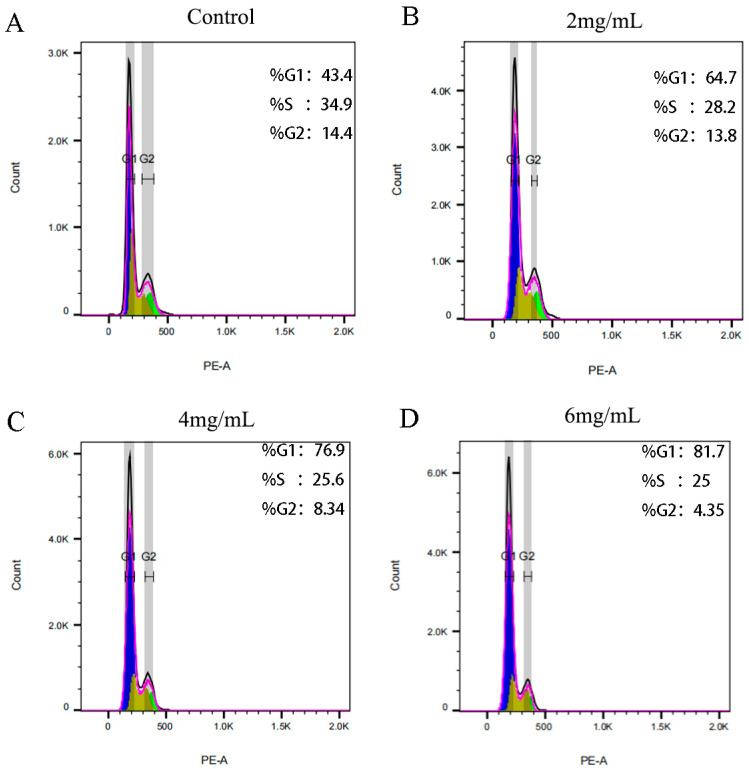
*RTDP* effect on the cell cycle of prostate cancer DU145 cells: (**A**) 0 mg/mL *RTDP* (control group), (**B**) 2 mg/mL *RTDP*, (**C**) 4 mg/mL *RTDP*, (**D**) 6 mg/mL *RTDP*. Purple, yellow, and green represent the G0/G1 phases, S phases, and G2/M phases of the cell cycle. (PE-A represents the fluorescence intensity, which is the dead cells contained under the fluorescence signal curve).

The impact of *RTDP* on the cell cycle of DU145 prostate cancer cells was assessed using propidium iodide staining, with results detailed in Figure 13. Following a 48 h treatment with various concentrations of *RTDP*, the cell cycle distributed in G0/G1 phase was as follows: 48.87% ± 4.73% in the control group; 65.57% ± 1.59% (*p* < 0.01) in the 2 mg/mL *RTDP* group; 76.47% ± 1.02% (*p* < 0.01) in the 4 mg/mL *RTDP* group; and 79.2% ± 2.18% (*p* < 0.01) in the 6 mg/mL *RTDP* group. These findings indicate an elevation in the percentage of DU145 cells at the G0/G1 phase with increasing *RTDP* doses. Comparative analysis among the *RTDP*-treated groups revealed that the 6 mg/mL *RTDP* group exhibited the most pronounced cell cycle arrest at the G0/G1 phase, suggesting that *RTDP* induces a dose-dependent cell cycle blockade of human prostate cancer DU145 cells at the G0/G1 phase (Figure 13).

#### 2.6.5. Induction of Apoptosis in DU145 Cells by RTDP

**Figure 14:**
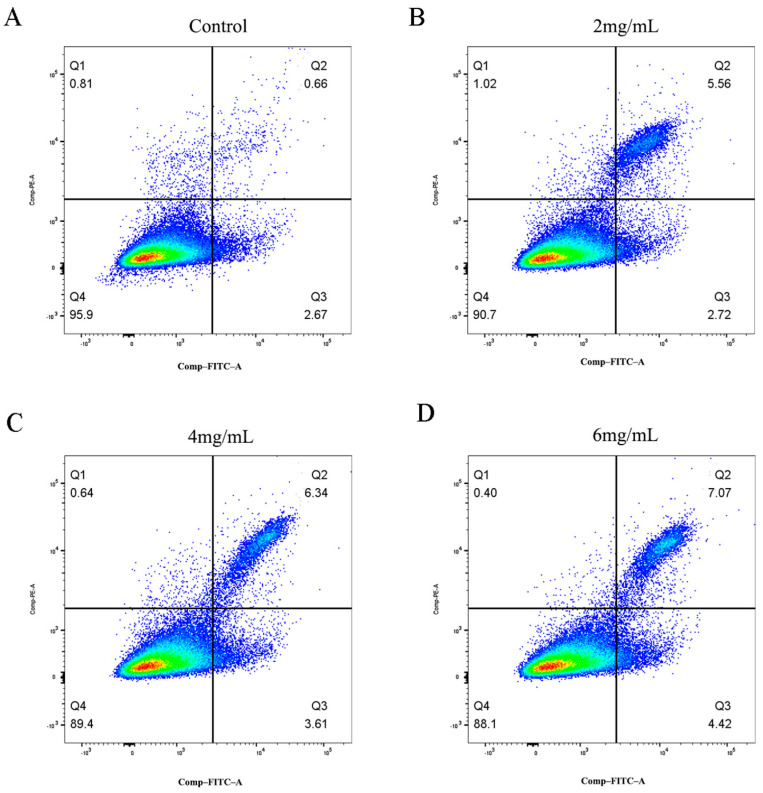
*RTDP* effect on the apoptosis of prostate cancer DU145 cells: (**A**) 0 mg/mL *RTDP* (control group), (**B**) 2 mg/mL *RTDP*, (**C**) 4 mg/mL *RTDP*, (**D**) 6 mg/mL *RTDP*. (Q1, Q2, Q3, and Q4 regions represent necrotic cells, late apoptotic cells, early apoptotic cells, and living cells, respectively).

The influence of *RTDP* on the apoptosis of DU145 cells was assessed using the Annexin V-FITC/PI assay, with results presented in Figure 14. In comparison to the control group (3.4% ± 0.37%), the apoptosis rate of DU145 cells treated with *RTDP* for 48 h was significantly elevated: 5.73% ± 0.98% (*p* < 0.05) in the 2 mg/mL group; 8.58% ± 0.27% (*p* < 0.01) in the 4 mg/mL group; and 11.03% ± 0.94% (*p* < 0.01) in the 6 mg/mL group. The apoptosis rate of DU145 cells escalated in a dose-dependent manner with increasing *RTDP* concentrations.

#### 2.6.6. RTDP Effect on the Expression of Cell Cycle and Apoptosis-Related Proteins in Prostate Cancer DU145 Cell Assessed by Western Blotting

**Figure 15:**
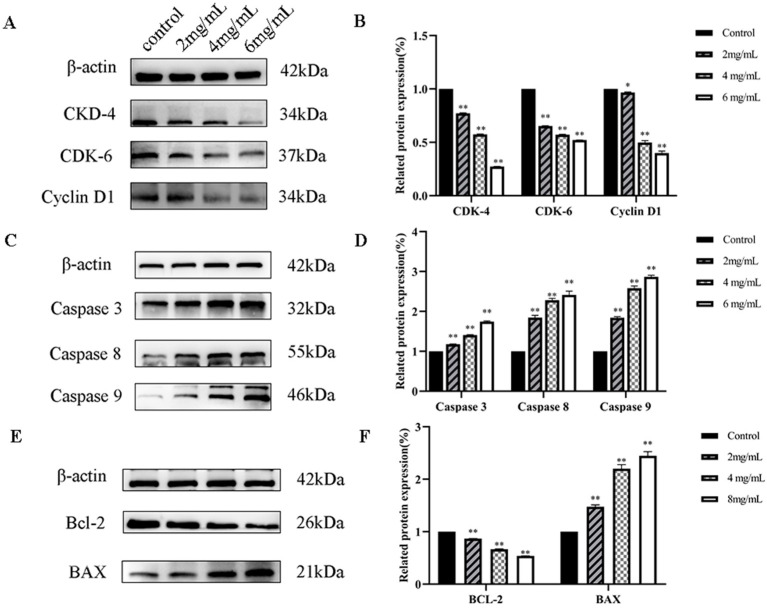
Effect of *RTDP* on prostate cancer DU145 cells. (**A**,**B**) Cyclin-dependent kinases (CDK-4 and CDK-6), as well as Cyclin D1 proteins of DU145 cells affected by *RTDP*; (**C**,**D**) pro-apoptotic proteins Caspase 3, Caspase 8, and Caspase 9 of DU145 cells affected by *RTDP*; (**E**,**F**) Bcl-2 family members Bcl-2 and Bax of DU145 cells affected by *RTDP*. All values are represented as mean ± SD (n = 3). ** *p* < 0.01, * *p* < 0.05 compared with the control (0 mg/mL).

Figure 15 illustrates that when DU145 cells were subjected to various concentrations of *RTDP* for 48 h, the expression levels of cyclin-dependent kinase (CDK)-4 and CDK-6, along with Cyclin D1, were significantly diminished across all *RTDP* groups compared to the control group. This reduction became more pronounced with increasing *RTDP* concentration. Specifically, when the *RTDP* concentration exceeded 2 mg/mL, the reductions in expression levels of CDK-4, CDK-6, and Cyclin D1 were significant (*p* < 0.01). Concurrently, with escalating *RTDP* doses, there was a notable elevation in the expressions of pro-apoptotic proteins Caspase 3, Caspase 8, and Caspase 9. Compared to the control group, the apoptosis proteins were significantly upregulated in DU145 cells treated with different *RTDP* doses (*p* < 0.01). Additionally, the expression of Bcl-2 was progressively downregulated, while BAX expression was up-regulated, both showcasing significant alterations in protein expression levels. In summary, potential therapeutic effect of *RTDP* against prostate cancer may be attributed to its ability to disrupt the cell cycle and induce apoptosis in DU145 cells, suggesting a concentration-dependent mechanism (*p* < 0.05) in the comparative analysis across *RTDP*-treated groups.

#### 2.6.7. The Effects of RTDP on the Expression of DU145 Cell Cycle and Apoptosis-Related Genes Detected by Quantitative Real-Time PCR (RT-qPCR)

**Figure 16:**
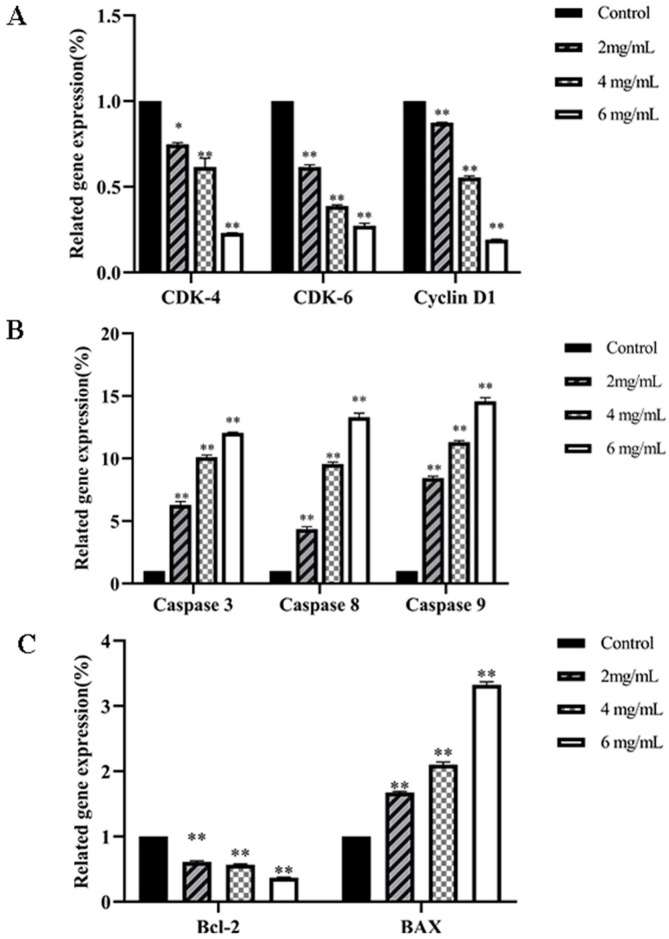
*RTDP* effect on cell-cycle-related mRNAs in DU145 cells. (**A**) Effect of *RTDP* on genes CDK-4, CDK-6, and Cyclin D1 of DU145 cells; (**B**) effect of *RTDP* on pro-apoptotic genes Caspase 3, Caspase 8, and Caspase 9 of DU145 cells; (**C**) effect of *RTDP* on genes Bcl-2 and Bax of DU145 cells. All values are represented as mean ± SD (n = 3). ** *p* < 0.01, * *p* < 0.05 compared with the control (0 mg/mL).

Figure 16 illustrates that in DU145 cells treated with varying *RTDP* concentrations for 48 h, clls mRNA expressions of Caspase 3, Caspase 8, Caspase 9, and Bax were upregulated in a dose-dependent manner compared to the control group (*p* < 0.01). Conversely, within the *RTDP*-treated group, mRNA levels of the cell-cycle-related genes CDK-4, CDK-6, and Cyclin D1 decreased significantly (*p* < 0.01), while Bcl-2 expression was notably inhibited (*p* < 0.05). These results indicate that *RTDP* significantly upregulates pro-apoptotic genes and downregulates cell-cycle-related genes in DU145 prostate cancer cells. Furthermore, the magnitude of *RTDP*’s effects on DU145 cells positively correlated with *RTDP* concentration and treatment duration, suggesting a concentration- and time-dependent inhibitory action of *RTDP* on the proliferation of DU145 prostate cancer cells (*p* < 0.05).

## 3. Materials and Methods

### 3.1. Instruments

The FST III-20 ultrapure water machine was purchased from purifier Co., Ltd. (Shanghai, China).; B type universal mill was purchased from Baokang Drying Machinery Co., Ltd. (Changzhou, China); thermostatic water bath was purchased from Boxunda Industrial Co., Ltd. (Shanghai, China); BT-224S electronic analytical balance was purchased from Sartorius Instrument Co., Ltd. (Beijing, China); SP-722 Ultraviolet–Visible spectrophotometer was purchased from Spectrum Instruments Co., Ltd. (Shanghai, China); freeze dryer was purchased from Bo Medical Health Technology Co., Ltd. (Beijing, China); KDC-140HR high-speed refrigerated centrifuge was purchased from Zhongke Zhongjian Scientific Instrument Co., Ltd. (Anhui, China); HF212 cell incubator was purchased from Shenli Scientific Instrument Co., Ltd. (Shanghai, China); MB-530 multi-function microplate reader was purchased from Huisong Technology Development Co., Ltd. (Shenzhen, China); CKX53 biological microscope was purchased from Olympus Corp. (Tokyo, Japan); BD FACSCanto II Flow cytometer was purchased from Becton Dickinson, (Franklin Lake, NJ, USA); StepOne Real-Time PCR System was purchased from Thermo Fisher Scientific (Rockford, IL, USA).

### 3.2. Reagents

*Rosa roxburghii* fruit was sourced from the Chinese herbal medicine wholesale market in Dali Bai Autonomous Prefecture, Yunnan Province, and authenticated by Dr. G.Y. Chen, a professor at Dali University. Monosaccharide standards, comprising arabinose (Ara), fructose (Fru), ribose (Rib), fucose (Fuc), glucose (Glc), mannose (Man), galactose (Gal), xylose (Xyl), galacturonic acid (GalA), and glucuronic acid (GlcA), were purchased from Sigma Chemical Co. (St. Louis, MO, USA). A series of dextran with varied molecular weights was purchased from the National Institutes for Food and Drug Control (Beijing, China), with all reagents meeting analytical reagent purity specifications. DU145 cells were procured from the Shanghai CAS Cell Bank (Shanghai, China). Dulbecco’s Modified Eagle Medium (DMEM) and fetal bovine serum (FBS) were purchased from Procell Life Technology Co., Ltd. (Wuhan, China), and enhanced CCK-8 reagent was purchased from APE×BIO Biotech (Suzhou, China). A bicinchoninic acid (BCA) protein concentration determination kit was purchased from Solarbio Technology Co., Ltd. (Beijing, China). A polyacrylamide gel electrophoresis (PAGE) gel kit was purchased from Epizyme Biomedical Technology Co., Ltd. (Shanghai, China). An ECL chemiluminescence kit was purchased from Share-bio Technology Co., Ltd. (Shanghai, China), and a NovoScript Plus All-in-one 1st Strand cDNA Synthesis SuperMix kit and a NovoStart SYBR qPCR SuperMix Plus kit were purchased from Novoprotein Technology Co., Ltd. (Suzhou, China).

### 3.3. Methods

#### 3.3.1. Extraction and Decoloration of *Rosa roxburghii* Tratt Polysaccharides

The dried powder of *Rosa roxburghii* Tratt was extracted and refluxed, with degreasing achieved through successive washes with petroleum ether (at a volume ratio of three times that of the powder), followed by treatment with 80% ethanol (for two cycles, each lasting 4 h). Polysaccharides were then extracted using pressurized hot water, followed by centrifugation to separate the extract. Subsequently, the solution was concentrated under reduced pressure, followed by precipitation with alcohol. The resulting precipitate underwent centrifugation and sequential washing with anhydrous ethanol, acetone, and ether. Removal of pigments was accomplished using resin, while proteins were eliminated employing the Sevag method [35]. Subsequent freeze-drying yielded the preliminary refined *Rosa roxburghii* Tratt decolorized polysaccharide (*RTDP*).

#### 3.3.2. Isolation and Purification of the Polysaccharides

The crude polysaccharide was dissolved in deionized water and subjected to purification using a DEAE-Sepharose FF column and a Sephadex G-100 column, with a slightly modified method [36]. Gradient elution was performed using NaCl solutions of varying concentrations (0 mmol/L, 0.1 mmol/L, 0.2 mmol/L, 0.3 mmol/L, 0.4 mmol/L, 0.5 mmol/L) and a flow rate of 0.001 L/min. The samples were monitored using the phenol–sulfuric acid method.

#### 3.3.3. Determination of Molecular Weight and Monosaccharide Composition Analysis

A standard solution with a concentration of 2.5 mg/mL was prepared and analyzed using High performance gel permeation chromatography (HPGPC). The chromatogram was recorded using a differential refractive index detector, and a standard curve correlating retention time to molecular weight was established. The retention time of the sample was plotted against the standard curve to determine its molecular weight, and data were analyzed using ASTRA 6.1.

Monosaccharide standards (Man, Ara, Rha, Glc, Gal, GlcUA, GalUA, GlcNAc, Fuc) were weighed and dissolved in deionized water, then diluted to create solutions with concentrations of 0, 10, 20, 50, 100, and 200 times the original concentration. A small amount (0.4 mg) of the sample was weighed and dissolved in 1 mL of deionized water to yield a 0.4 mg/mL sample solution. For hydrolysis, 1 mL of each mixed standard and sample solution was mixed with 4 mol/L trifluoroacetic acid (TFA) and subjected to hydrolysis in an oil bath at 110 °C for 4 h. Postreaction, the mixture was evaporated and dried. The hydrolyzed sample and monosaccharide standard solution were mixed with 400 μL of 0.5 mol/L PMP methanol solution, evenly mixed, and subjected to a derivatization reaction in an oven at 70 °C for 1 h, then cooled to room temperature. The mixture was neutralized with 0.3 mol/L HCl and extracted three times with CHCl_3_. The aqueous phase was centrifuged, filtered through a 0.22 μm microporous membrane, and subjected to analysis via HPLC.

#### 3.3.4. FT-IR and Ultraviolet–Visible Spectroscopy

Approximately 1 g of *RTDP* was accurately weighed and dissolved in 100 mL of deionized water, then scanned using a Ultraviolet–Visible spectrometer (UV–vis) (TU-1901, Persee General Instrument Co., Beijing, China) over the wavelength range of 200–400 nm. For FT-IR analysis, *RTDP* and dried KBr powder were ground at a ratio of 1:4, pressed into tablets, and examined using a Thermo Nicolet FT-IR spectrometer (Thermo Fisher Scientific Inc., Waltham, MA, USA) spanning the range of 500–4000 cm^−1^. For UV analysis, utilizing the Sevag method [37], the polysaccharide solution was treated with a chloroform and n-butanol reagent in a specific ratio. After reacting at room temperature for 20 min, samples were scanned in the 200–400 nm range.

#### 3.3.5. Congo Red Test

The conformational characteristics of the *RTDP* were assessed using a modified Congo red method [38]. Initially, 5 mg of *RTDP* was dissolved in 1 mL of deionized water along with 1 mL of Congo red reagent (80 μmol/L). Subsequently, incremental additions of 1 mol/L NaOH solution were made to achieve a final concentration gradient (0, 0.1, 0.2, 0.3, 0.4, 0.5 mol/L). The maximum absorption wavelength of the *RTDP*-Congo red complex under various NaOH concentrations was determined using a UV–vis within the wavelength range of 200–800 nm, facilitating the assessment of the presence or absence of a triple helix structure in *RTDP*.

#### 3.3.6. Preparation of the Glucose Standard Curve

Standardized glucose was accurately weighed and dissolved in water to prepare a 100 μg/mL standard glucose solution. Subsequently, 0.05, 0.10, 0.20, 0.30, 0.40, 0.50, 0.60, 0.70, 0.80, and 0.90 mL of this glucose standard solution were dispensed into 10 sterile test tubes, respectively. Each tube was then diluted to 2 mL with water, followed by the addition of 1 mL phenol solution and 5 mL concentrated sulfuric acid, then mixed and left at room temperature for 30 min. Absorbance (A) was measured at 490 nm and plotted against glucose concentration (C) to derive the standard curve regression equation:

Y=1.6578X+0.0141 R2=0.9994.

#### 3.3.7. Determination of the Extraction Yield of Polysaccharides from *Rosa roxburghii* Tratt

Absorbance was measured at 620 nm using DI water as the reference. The standard glucose curve was employed to calculate the mass concentration of *Rosa roxburghii* Tratt polysaccharides. The extraction rate was computed using the following equation:

$$Y=\frac{\rho \;\times \;V\;\times \;N}{m}\times 100\%$$

where *Y*: extraction yield of *Rosa roxburghii* Tratt polysaccharides; *ρ*: polysaccharide concentration; *V*: polysaccharide volume; *N*: dilution ratio; *m*: quality of raw material.

#### 3.3.8. Single-Factor Experiment on Water Extraction and Alcohol Precipitation

The impact of material-to-liquid ratio, extraction temperature, extraction time, and extraction frequency on the extraction rate of *Rosa roxburghii* Tratt polysaccharides was investigated, with all other parameters held constant.

### 3.4. Response-Surface Design for Polysaccharide Extraction

Building on the single-factor experiment, a Box–Behnken response surface design was constructed with four factors at three levels (Table 8) using Design-Expert 13, with the total polysaccharide yield (*Y*) designated as the target variable [39].

#### 3.4.1. Resin Pretreatment

FL-1 macroporous resin was immersed for 6 h in NaOH, HCl, and 95% ethanol, respectively. Then it was rinsed with deionized water until pH neutral. The resin was stored in a wet state for subsequent experiments.

#### 3.4.2. Pigment Removal from *Rosa roxburghii* Tratt Polysaccharides

The pretreated FL-I macroporous resin was combined with the *Rosa roxburghii* Tratt polysaccharide solution in a specified ratio, resulting in decolorized polysaccharides for further analysis.

#### 3.4.3. Determination and Calculation of Decolorization Rate

The polysaccharide solution mixed with phenol and sulfuric acid was scanned at 330–880 nm, the point of maximum absorbance, chosen for subsequent absorbance measurements. The decolorization rate was calculated using the following equation:

$$D=\frac{A_{0}-A_{1}}{A_{0}}\times 100\%$$

where *D*: decorating rate of *Rosa roxburghii* Tratt polysaccharides, *A*_0_: absorbency before decolorization, *A*_1_: absorbency after decolorization.

#### 3.4.4. Determination and Calculation of Polysaccharide Retention Rate

The mixed solutions of *RTDP*, phenol, and sulfuric acid was scanned at 490 nm, and the polysaccharide retention rate was calculated using the following equation:

$$R=\left[1-\frac{A_{0}-A_{1}}{A_{0}}\right]\times 100\%$$

where *R*: retention rate of *Rosa roxburghii* Tratt polysaccharides, *A*_0_: absorbency before decolorization, *A*_1_: absorbency after decolorization.

#### 3.4.5. Comprehensive Score

To evaluate the decolorization effect, two indices were considered: the rate of polysaccharide pigment removal and the rate of polysaccharide retention. A weighted scoring method was applied to assess the decolorization effect. Weight coefficients for the decolorization rate (*X*) and polysaccharide retention rate (*Y*) were both set at 0.5. The comprehensive score (*S*) was then weighted and combined as follows:

S=0.5X+0.5Y

#### 3.4.6. Single-Factor Test

Polysaccharide solutions of 5 mg/mL were prepared, and keeping all other factors constant, the effects of decoloration time (1.0, 2.0, 3.0, 4.0, 5.0 h), resin content (1.0, 2.0, 3.0, 4.0, 5.0 g), and decoloration temperature (30, 40, 50, 60, 70 °C) were assessed on both the rate of polysaccharide pigment removal and polysaccharide retention.

### 3.5. Response Surface Test Design for the Decolorization of Polysaccharide

Based on the single-factor tests, decolorization time (*A*), decolorization temperature (*B*), and resin content (*C*) were chosen as the variables for a three-factor, three-level (Table 7) response surface methodology aimed at optimizing the decolorization technology. The comprehensive score (*S*), which encompasses both the decolorization and retention rates, served as the response variable.

### 3.6. The Antioxidant Activity of Rosa roxburghii Tratt Polysaccharide

Antioxidant capacity serves as a pivotal link and bridge connecting various biological activities. Oxidative stress represents a prominent characteristic of tumor cells, extending throughout the entirety of tumor development. Excessive oxidative stress has the potential to induce oxidative damage to biological macromolecules within cells. Subsequently, the capacity of *RTDP* and Vitamin C (VC) to scavenge OH^−^ ions under identical conditions was compared subsequent to their separate mixing with the OH^−^ solution.

### 3.7. Cell Culture

DU145 cells were cultured using DMEM supplemented with 10% FBS and penicillin/streptomycin, in an incubator with 37 °C and 5% CO_2_. Prostatic cancer DU145 cells grown to the logarithmic phase were collected and subcultured.

#### 3.7.1. Cell Treatment

A specified amount of *RTDP* was dissolved in DMEM, filtered twice using a disposable sterile filter, and administered to the DU145 cells at predetermined concentrations for further experimentation.

#### 3.7.2. Cell Viability Assessment via CCK-8 Assay

DU145 cells in the logarithmic growth phase were seeded at a density of 7 × 10^3^ cells/well in 100 μL of culture medium into 96-well plates, and cells were divided into Control, Blank, and *RTDP* groups. The Blank and *RTDP* groups received 100 μL of cell suspension, while the Control group was supplemented with 100 μL of DMEM. Upon cell adhesion, the initial medium was discarded, and 100 μL of DMEM medium was added to the Blank and Control groups. The *RTDP* group was exposed to various concentrations of *RTDP* in DMEM medium, achieving final concentrations of 2, 4, 6, 8, and 10 mg/mL. After incubation at 24, 48, and 72 h, the CCK-8 reagent at a 1:9 ratio was added to each well, followed by incubation for 90 min. Absorbance at 450 nm was measured and recorded. The 50% inhibiting concentration (IC50) were calculated, and cell viability was determined using the following equation:

$$V=\left[\frac{{OD}_{R}-{OD}_{B}}{{OD}_{C}-{OD}_{B}}\right]\times 100\%$$

where *V*: The viability of DU145 cells, *R*: The viability of DU145 cells in RTDP group, *B*: The viability of DU145 cells in blank group, *C*: The viability of DU145 cells in control group.

#### 3.7.3. Migration Rate Assessment via Scratch Assay

DU145 cells were seeded at a density of 7 × 10^6^ cells/plate into 6-well plates. The cells were categorized into Control, Blank, and *RTDP* groups. After cell density exceeded 95%, the medium was replaced with corresponding solutions. The migration of DU145 cells in the same position was recorded at 0, 24, and 48 h. The cell migration rate was calculated using the following equation:

$$M=\left[1-\frac{C-R}{C}\right]\times 100\%$$

where *M*: The migration rate of DU145 cells, *C*: The migration rate of DU145 cells in control group *R*: The migration rate of DU145 cells in RTDP group.

#### 3.7.4. Invasiveness Rate of DU145 Cells Assessed by Transwell Assay

DU145 cells were seeded in the upper chambers of 24-well plates at a density 6 × 10^4^ cells per 200 μL. Subsequently, 800 μL of DMEM medium was added to the lower chambers, and the assembly was incubated in a CO_2_ incubator for 8–12 h, after which the medium in the upper chamber was replaced with the predetermined concentration of *RTDP* for another 48 h. The cells were fixed with 4% paraformaldehyde for 20 min, stained with crystal violet for 15 min, and rinsed twice more with PBS. Cell invasion was recorded. The invasiveness rate was calculated with the following equation:

$$I=\left[1-\frac{C-R}{C}\right]\times 100\%$$

where *I*: The invasiveness rate of DU145 cells, *C*: The invasiveness rate of DU145 cells in control group, *R*: The invasiveness rate of DU145 cells in RTDP group.

#### 3.7.5. Cell Cycle Analysis of DU145 Cells by Flow Cytometry

DU145 cells were seeded in 6-well plates at a density of 7 × 10^7^ cells in 2 mL of medium. After 8–12 h, the medium was replaced with serum-free DMEM medium to induce cells starved for 24 h. Subsequently, medium containing various concentrations of *RTDP* was added and incubated for 48 h. The cells were fixed with 75% cold ethanol overnight at 4 °C, and stained with propidium iodide solution before analysis using a BD FACSCanto II Flow cytometer and FlowJo 10.8.1 [40].

#### 3.7.6. Apoptosis Detection in DU145 Cells by Flow Cytometry

DU145 cells were seeded in 6-well plates at a density of 7 × 10^7^ cells in 2 mL of medium and cultured for 8–12 h. Subsequently, DMEM medium containing various concentrations of *RTDP* was introduced, and the cells were treated for 48 h. Cells were collected and resuspended with Annexin V-FITC staining solution. Apoptosis rates in DU145 cells were assessed using a BD FACSCanto II Flow cytometer and FlowJo 10.8.1 [41].

#### 3.7.7. Detection of Cell Cycle and Apoptosis-Related Proteins in DU145 Cells by Western Blotting

After DU145 cells were treated with *RTDP* for 48 h, their proteins were collected for protein quantification by the BCA method to ensure equal protein loading in each group. A 10% SDS-PAGE gel was prepared for protein separation by electrophoresis, with the gel cut according to molecular weight markers. The separated proteins were transferred onto a PVDF membrane. The membrane was incubated with the primary antibody for 12 h and with the secondary antibody for 1 h. ECL chemiluminescence developer was mixed and applied to the membranes, and results were recorded in the imaging system. Image J 2023 was utilized to analyze the protein intensities.

#### 3.7.8. Analysis of Cell Cycle and Apoptosis-Related Proteins in DU145 Cells by Quantitative Real-Time PCR

DU145 cells were collected for RNA quantification using a Total assay, and the concentration and integrity of RNA were assessed. The RNA was reverse-transcribed into cDNA by the polymerase chain reaction (PCR) method, then by Quantitative real-time PCR employing SYBR qPCR Supermix Plus reagent and specific primers. The expressions of genes in DU145 cells were quantified using the 2^−∆∆Ct^ method.

### 3.8. Statistical Analysis 

All the above experiments were performed in triplicate, and the resulting data were analyzed using Design-Expert 13, Origin 2022, GraphPad Prism 9.5, Image J 2023, Adobe Photoshop 2021, and FlowJo 10.8.1. The measurement data are expressed as mean ± SD (*x* ± *s*). Results with *p*-values < 0.05 was considered statistically significant (* *p* < 0.05: significant difference; ** *p* < 0.01: highly significant difference). Analysis of variance (ANOVA) was used to assess the significance of differences among two or more samples, and *t*-tests were utilized to determine significant differences between the means of independent samples.

## 4. Discussion

Polysaccharides, polymeric carbohydrate molecules comprising elongated chains of monosaccharide units, represent natural biological macromolecules that have garnered considerable interest due to their multifaceted biological activities [42]. Numerous studies have delved into the extraction of polysaccharides from plants, elucidating their broad spectrum of biological functionalities. For instance, a polysaccharide fraction (PFCM) extracted from *Passiflora edulis* via hot water extraction exhibited remarkable inhibition of tumor growth in mice bearing Sarcoma 180 tumors [43]. Similarly, polysaccharides extracted from *Asparagus officinalis* using ultrasonic cycle extraction technology demonstrated anti-HeLa cell activity in vitro [44]. Additionally, the discovery of a novel heteropolysaccharide (CSP-W-2) derived from the fruit of *Chaenomeles speciosa* (Sweet) Nakai showcased its efficacy in suppressing HepG2 cell growth through the induction of nucleus shrinkage and cell apoptosis [45]. A water-soluble heteropolysaccharide, RMP1, possessing a molecular weight of approximately 137 kDa, was isolated from mulberry *Morus alba* twigs (ramulus mori), demonstrating its capacity to induce apoptosis and cell cycle arrest at the S phase in SGC-7901 cells [46]. Zeng investigated the extraction and antioxidant activity of polysaccharides from *Allium sativum*, showcasing their ability to scavenge superoxide anions and hydroxyl radicals [47]. Lastly, polysaccharides derived from pumpkin, extracted using hot water and subsequently chemically modified to yield phosphorylated variants (PP1, PP2), exhibited significant scavenging effects on superoxide anions and hydroxyl radicals, as assessed through pyrogallol autooxidation and the salicylic acid method, respectively [48].

Located in southwest China, Yunnan Province benefits from a warm climate with notable diurnal temperature fluctuations, presenting distinctive conditions conducive to the cultivation of natural plants. Numerous bioactive compounds found in these plants have demonstrated antioxidant, hypoglycemic, anti-tumor, and anti-inflammatory properties [49,50,51,52]. Plant polysaccharides, as active ingredients extracted from these plants, have garnered research interest due to their non-toxic nature and affordability. Extensive research has highlighted the therapeutic potential of polysaccharides, positioning them as valuable additives in food and nutritional products [53,54,55], thus augmenting their commercial value.

*Rosa roxburghii* Tratt, a member of the family Rosaceae, represents a significant botanical resource widely distributed throughout southwest China, esteemed for its remarkable nutritional and health-enhancing attributes. It holds a distinguished status as both a traditional edible and medicinal plant within Chinese culture. Recent studies have unveiled an expanding array of bioactive components in *R. roxburghii*, highlighting its healthcare and medicinal potential [56]. Notably, *R. roxburghii* stands out for possessing the highest vitamin C content among all fruits, earning it the title of “king of Vitamin C” [57]. The genus *Rosa*, constituting approximately 200 species worldwide, within the family Rosaceae, showcases significant medicinal and nutritional properties [58]. A neutral water-soluble polysaccharide (RLP50-2) extracted from *Rosa laevigata* fruits exhibited notable anti-tumor efficacy by inhibiting K562 cell proliferation and migration while blocking angiogenesis [59]. Research on *R. roxburghii* identified pentacyclic triterpene acid as its principal constituent, which activates the ROS/JNK signaling pathway, leading to the arrest of liver cancer cell proliferation at the G2/M phase and induction of apoptosis via the mitochondrial pathway [60]. *R. roxburghii* has been demonstrated to ameliorate insulin resistance in obese rats through the induction of antioxidant stress and augmentation of the expressions of PI3K, Akt2, and GLUT4 proteins and genes [61]. Furthermore, a hydroalcoholic extract derived from *R. roxburghii* fruit (HRT), enriched with phenolic acids, exhibited significant reductions in body weight and lipid levels in hyperlipidemic rats [62]. Flavonoids extracted from *R. roxburghii* (FRRT) have shown protective effects on cardiomyocytes against DOX-induced contraction and autophagy [63]. Therefore, the exploration of novel polysaccharides with anticancer and immunopotentiating activities from natural sources holds promise for innovative approaches in cancer therapy [64]. 

*Rosa roxburghii* Tratt fruits were repeatedly dried to eliminate moisture interference. According to single-factor experiment and response surface methodology (Table 8 and Figure 1), polysaccharides were extracted through a process involving hot water extraction followed by alcohol precipitation. Initially, dried fruits were finely ground and sieved through a 0.18 mm mesh to enhance solubility in water. A specific quantity of *R. roxburghii* dry powder was then dissolved in a conical flask to a constant volume. Ultrasonic waves were employed to dislodge any powder adhering to the bottom of the flask. The solution of *R. roxburghii* was mixed with absolute ethanol in a constant temperature water bath to precipitate the polysaccharides. Subsequently, ethanol was recovered, retaining the polysaccharide solution. The presence of proteins and nucleic acids in the polysaccharide solution was assessed at 260–280 nm using an ultraviolet spectrophotometer.

The polysaccharide solution underwent a decolorization process with D-101 adsorbent resin under specified conditions. Post-decolorization, the solution’s color transitioned from reddish-brown to clear light yellow. Proteins were removed from the decolorized solution using the Sevag method, followed by alcohol precipitation and rotary evaporation to yield a concentrated solution. This concentrated solution was freeze-dried at −40 °C to obtain the preliminarily purified polysaccharide (*RTDP*), with protein and nucleic acid assessments conducted at 260–280 nm using an ultraviolet spectrophotometer (Figure 4B). Further purification of this polysaccharide was conducted using DEAE-52 cellulose and Sephadex G-100 column chromatography for subsequent analyses (Figure 2). The characteristic functional groups of *RTDP* were identified via FT-IR, and its molecular weight was determined through gel chromatography to evaluate polysaccharide homogeneity (Figure 3A). Following acid hydrolysis and derivatization, the monosaccharide composition of *RTDP* was analyzed using HPLC. The analysis revealed *RTDP* to be an acidic polysaccharide, featuring a carboxyl group, with monosaccharide molar ratios of 5.93% D-mannose, 3.77% L-rhamnose, 1.76% N-acetyl-D-glucosamine, 1.50% D-galacturonic acid, 39.87% D-glucose, 20.47% D-galactose, 1.94% D-xylose, 12.93% L-arabinose, and 11.83% L-fucose (Figure 3B).

This study confirmed that *RTDP* may induce apoptosis in DU145 cells by concurrently modulating the death receptor and mitochondrial pathways, consequently impeding the progression of prostate cancer. This research provides a foundation for future investigations to delineate the specific pathways through which *RTDP* inhibits DU145 cell proliferation in vivo, thus advancing the comprehension of *RTDP*’s anti-prostate cancer effects (Supplementary Materials).

## 5. Conclusions

In the present research, polysaccharides (RTDP) were isolated and purified from Yunan rose *Rosa roxburghii* Tratt using water extraction and alcohol precipitation methods. The DEAE-52 cellulose chromatography column and Sephadex G-100 gel column facilitated the separation and purification of these polysaccharides. The molecular weight of RTDP is approximately 2.7 × 10^2^ kDa. Analysis results showed that RTDP is an acidic polysaccharide with a triple helix structure, and glucose was the most abundant monosaccharide in RTDP (Figure 3B and Figure 5). Characterization was performed using ultraviolet spectroscopy (UV), infrared spectroscopy (FT-IR), and high-performance liquid chromatography (HPLC). The antioxidant activity of the *Rosa roxburghii* Tratt polysaccharides was evaluated using hydroxyl radical (OH^−^) scavenging assays, which indicated that RTDP has good antioxidant activity in vitro. In addition, RTDP inhibited the proliferation, migration, and invasion of DU145 cells. By up-regulating the cycle-related factors CDK-4, CDK-6, and Cyclin D1 and down-regulating the apoptosis-related factors Caspase 3, Caspase 8, Caspase 9, and BAX, RTDP can arrest the cell cycle at G0/G1 phase and induce apoptosis, indicating that RTDP has significant anti-prostate cancer DU145 cells activity.

The results demonstrated that *RTDP* significantly reduced the survival rate of DU145 cells and inhibited their migration and invasion abilities. *RTDP* was also observed to upregulate the expression of apoptosis-related genes and proteins, including Caspase 3, Caspase 8, and Caspase 9 in DU145 cells, indicating potential cell cycle arrest at the G0/G1 phase. Furthermore, the expression levels of cell cycle-associated genes and proteins such as CDK-4, CDK-6, and Cyclin D1 were reduced, with their suppression positively correlated with *RTDP* concentration and exposure duration. Additionally, the BCL-2/BAX protein ratio exhibited a progressive increase following *RTDP* treatment, signifying enhanced apoptosis in DU145 cells. Collectively, these findings suggest that *RTDP* may prevent prostate cancer progression by inhibiting DU145 cell proliferation and inducing apoptosis through cell cycle arrest. The results suggest that RTDP may be an ideal natural antioxidant and a potential therapeutic agent for prostate cancer.

## Figures and Tables

**Table 1:** Box–Behnken test design and results.

Serial Numbers	*A*	*B*	*C*	*D*	*Y*/% (Extraction Yield)
1	−1	1	0	0	25.64
2	0	0	1	1	24.19
3	1	0	0	−1	26.96
4	0	0	−1	1	26.94
5	−1	0	1	0	25.97
6	0	0	0	0	29.85
7	−1	0	0	1	24.29
8	0	−1	0	−1	27.46
9	0	−1	−1	0	26.34
10	1	0	−1	0	27.45
11	0	0	0	0	29.74
12	0	0	−1	−1	27.24
13	0	−1	0	1	25.62
14	1	−1	0	0	27.53
15	1	0	1	0	25.32
16	0	1	1	0	27.15
17	0	0	0	0	28.95
18	0	1	0	1	25.98
19	1	1	0	0	28.28
20	0	1	0	−1	28.72
21	0	0	1	−1	26.55
22	0	0	0	0	29.84
23	0	1	−1	0	30.56
24	−1	0	0	−1	26.35
25	−1	−1	0	0	24.73
26	0	−1	1	0	25.38
27	0	0	0	0	29.18
28	−1	0	−1	0	26.89
29	1	0	0	1	24.58

**Table 2:** Response-surface ANOVA results.

Source	Sum of Squares	df	Mean Square	F Value	*p*-Value Prob > F	Significance
Model	80.96	14	5.78	10.18	<0.0001	**
*A*	6.31	1	6.31	11.11	0.0049	**
*B*	7.16	1	7.16	12.61	0.0032	**
*C*	12.06	1	12.06	21.24	0.0004	**
*D*	11.37	1	11.37	20.02	0.0005	**
*AB*	0.0064	1	0.0064	0.011	0.9170	
*AC*	0.18	1	0.18	0.31	0.5864	
*AD*	0.026	1	0.026	0.045	0.8349	
*BC*	1.5	1	1.5	2.64	0.1263	
*BD*	0.2	1	0.2	0.36	0.5599	
*CD*	1.06	1	1.06	1.87	0.1932	
*A* ^2^	30.1	1	30.1	53.01	<0.0001	**
*B* ^2^	3.28	1	3.28	5.77	0.0307	*
*C* ^2^	11.81	1	11.81	20.79	0.0004	**
*D* ^2^	20.56	1	20.56	36.21	<0.0001	**
Residual	7.95	14	0.57			
Lack of Fit	7.25	10	0.72	4.14	0.0915	non-significant
Pure Error	0.7	4	0.17			
Cor Total	88.91	28				

*R*^2^ = 0.9106; *R*^2^_Adj_ = 0.8212 (** *p* < 0.01, * *p* < 0.05).

**Table 3:** Statistical analysis of the error of the regression model.

Item	Value	Item	Value
Std.Dev.	0.7535	R-Squared	0.9106
Mean	27.02	Adjusted R-Squared	0.8212
C.V./%	2.79	Pred R-Squared	0.5058
PRESS	43.94	Adequate Precision	11.3

**Table 4:** Design and outcomes of the response surface experiment.

Serial Number	*A*	*B*	*C*	Decolorizing Rate (%)	Retention Rate (%)	Comprehensive Score (*S*)
1	−1	−1	0	21.45	84.54	57.00
2	1	−1	0	71.55	66.48	61.82
3	−1	1	0	44.49	83.11	63.8
4	1	1	0	77.2	49.36	63.28
5	−1	0	−1	12.34	89.55	57.95
6	1	0	−1	57.98	67.27	62.63
7	−1	0	1	64.44	63.67	64.06
8	1	0	1	87.38	27.58	61.58
9	0	−1	−1	23.29	80.07	51.68
10	0	1	−1	31.74	75.22	58.48
11	0	−1	1	62.66	57.35	60.01
12	0	1	1	80.55	30.71	55.63
13	0	0	0	76.56	67.23	73.35
14	0	0	0	76.89	68.59	72.74
15	0	0	0	73.91	71.45	72.68
16	0	0	0	71.58	70.46	71.02
17	0	0	0	74.64	69.85	72.25

**Table 5:** Variance results of the response-surface analysis.

Source	Sum of Squares	df	Mean Square	F Value	*p* Value	Significance
Model	711.71	9	79.08	70.43	<0.0001	**
*A*	5.28	1	5.28	4.7	0.0667	
*B*	14.26	1	14.26	12.7	0.0092	**
*C*	13.89	1	13.89	12.37	0.0098	**
*AB*	7.13	1	7.13	6.35	0.0398	*
*AC*	12.82	1	12.82	11.41	0.0118	*
*BC*	31.25	1	31.25	27.83	0.0012	**
*A* ^2^	35.75	1	35.75	31.84	0.0008	**
*B* ^2^	270.76	1	270.76	241.14	<0.0001	**
*C* ^2^	265.38	1	265.38	236.36	<0.0001	**
Residual	7.86	7	1.12			
Lack of fit	4.84	3	1.61	2.13	0.2388	non-significant
Pure error	3.02	4	0.76			
Cor total	719.57	16				

*R*^2^ = 0.9891; *R*^2^_Adj_ = 0.9750 (** *p* < 0.01, * *p* < 0.05).

**Table 6:** Statistical analysis of the error from regression model.

Item	Value	Item	Value
Std.Dev.	1.06	R-Squared	0.9891
Mean	63.53	Adjusted R-Squared	0.9750
C.V./%	63.53	Pred R-Squared	0.8859
PRESS	82.11	Adequate Precision	26.339

**Table 7:** Test factors and levels of response-surface design.

Levels	Factors		
	*A* Time (h)	*B* Temperature (°C)	*C* Resin Content (g)
−1	2	40	2.0
0	3	50	3.0
1	4	60	4.0

**Table 8:** Response-surface factors and levels.

Level	*A* Extraction Time (h)	*B* Extraction Temperature (°C)	*C* Material–Liquid Ratio (g/mL)	*D* Extraction Frequency (Freq)
−1	2	50	1:20	2
0	3	60	1:30	3
1	4	70	1:40	4

## Data Availability

Data is contained within the article.

## Supplementary Materials

The following supporting information can be downloaded at: https://www.mdpi.com/article/10.3390/molecules29071575/s1.
