# Supplementary material for: Inhibition of Proliferation and Induction of Apoptosis in Prostatic Carcinoma DU145 Cells by Polysaccharides from Yunnan Rosa roxburghii Tratt
Source: Molecules. 2024 Apr 1;29(7):1575. doi: 10.3390/molecules29071575 (PMC11013296; doi:10.3390/molecules29071575)
Supplement: Supplementary file 1 [file molecules-29-01575-s001.zip › molecules-2797167-supplementary.pdf]

Supplementary Materials

1. Cell proliferation assay

RTDP was applied to normal liver cells LO2, and the proliferation inhibition of the cells was recorded after 48 h .

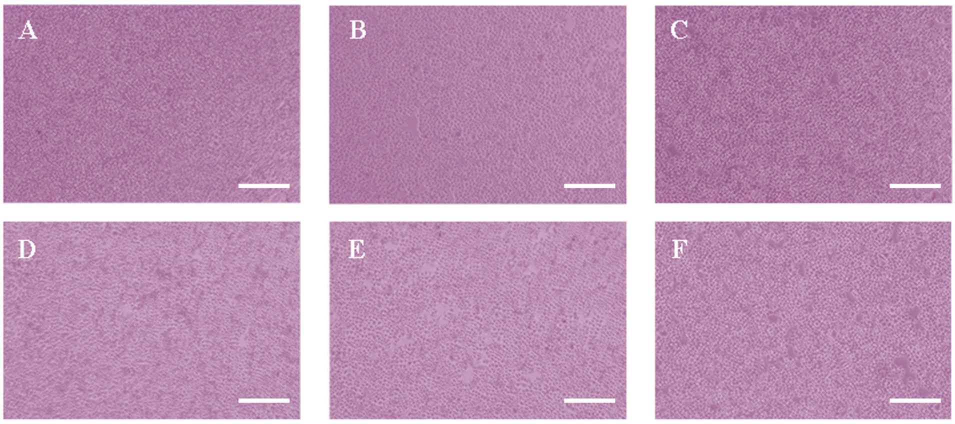

Figure S1. LO2 cells treated with RTDP for 48h (Scales 100 μm) A: RTDP 0mg/mL; B: RTDP 2mg/mL; C: RTDP 4mg/mL; D: RTDP 6mg/mL; E: RTDP 8mg/mL; F: RTDP 10mg/mL

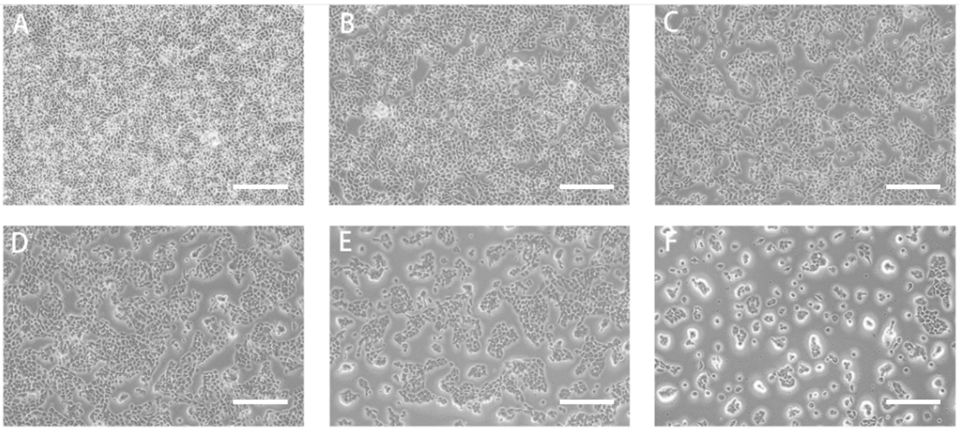

Figure S2. DU145 cells treated with RTDP for 48h (Scales 100 μm) A: RTDP 0mg/mL; B: RTDP 2mg/mL; C: RTDP 4mg/mL; D: RTDP 6mg/mL; E: RTDP 8mg/mL; F: RTDP 10mg/mL

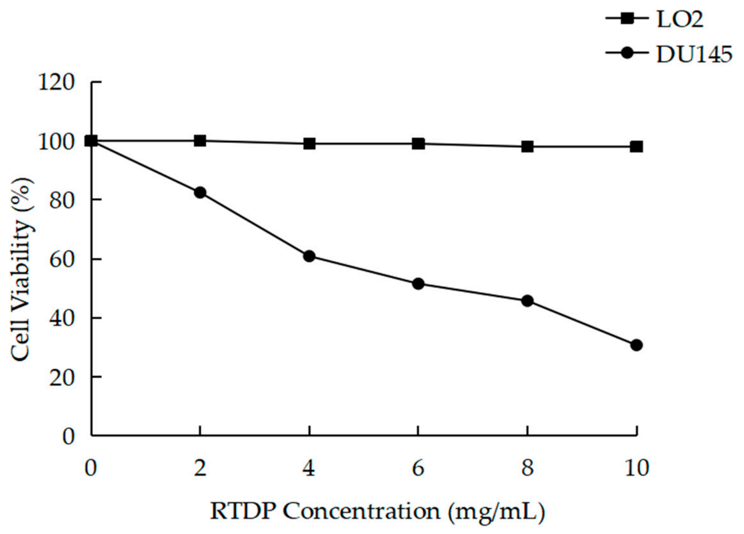

Figure S3. Viability of DU145 and LO2 cells treated with RTDP for 48h

Compared with DU145 cells, the inhibition of LO2 cell proliferation was significantly reduced, indicating that RTDP has no or very low toxicity to LO2 cells. These results suggest that RTDP has anti-tumor activity.

Table S1 Molar calculation of monosaccharides

monosaccharides	Molar ratio	Molar percentage（%）
D-Mannose	3.95	5.93
L-Rhamnose	2.51	3.77
N-Acetyl- glucosaminidase	1.17	1.76
D-Galacturonic Acid	1.00	1.50
D-Glucose	26.59	39.87
D-Galactose	13.65	20.47
D-Xyose	1.29	1.94
L-Arabinose	8.62	12.93
L-Fucose	7.89	11.83
